# Validation of the new “Brandenburg Acute Bile Duct (BABD) injury classification” system in 106 patients with accidental bile duct injuries during cholecystectomy

**DOI:** 10.1186/s13037-025-00451-1

**Published:** 2026-01-12

**Authors:** R. Mantke, J. Hafkesbrink, Paasch Ch, R. Hunger

**Affiliations:** 1 Brandenburg Medical School, Department of Surgery, University Hospital Brandenburg, Hochstrasse 29, 14770 Brandenburg, Germany; 2 Brandenburg Medical School, Faculty of Health Sciences, Brandenburg, Germany

**Keywords:** Bile duct injury, Laparoscopic cholecystectomy, Systematic classification

## Abstract

**Background:**

Bile duct injuries following laparoscopic cholecystectomy are rare but serious complications. Timely diagnosis and optimal management remain challenging. Classification systems for bile duct injuries may facilitate diagnosis, guide treatment, and improve outcomes, however their clinical use is limited. This study systematically evaluated existing classification systems and assessed their applicability to all types of injuries. Based on this analysis, a new classification system for acute lesions was developed.

**Methods:**

The database of the German Arbitration Board for medical liability issues was queried to identify cases involving bile duct injuries following a cholecystectomy (1990–2021). For each patient, the anatomical location, extent of injury, and therapeutic approach were documented. Injuries were categorized according to 11 published classification systems and a newly developed classification system. The ability to categorize bile duct injuries of all systems was assessed.

**Results:**

A total of 106 bile duct injuries were identified. The common bile duct was the most frequently injured structure (31.1%), followed by combined injuries (27.4%), and injuries of the common hepatic duct (17.9%). In 13.2% of cases, an artery was injured in addition to the bile duct lesion. Only 30.2% of bile duct injuries were detected intraoperatively. The most frequently performed techniques were biliodigestive anastomosis (34.4%), direct bile duct anastomosis (31.3%), and leakage closure with stitches (28.1%). None of the 11 existing classifications could categorize all cases; the best-performing systems (Amsterdam, Hannover) classified 82–86%. In contrast, the new Brandenburg Acute Bile Duct Injury (BABD) Classification was able to categorize 99% of the injuries.

**Discussion:**

Current classification systems fail to categorize all acute bile duct injuries following cholecystectomies. The BABD Classification, which is based on the anatomical location and extent of injury, allows systematic categorization of all documented acute bile duct lesions and may improve diagnostic clarity, treatment planning, and comparability in future studies.

## Introduction

Bile duct injuries, manifesting as bile leakage or stricture, are serious complications of laparoscopic cholecystectomy. Bile leaks are considered early complications, whereas strictures are typically late sequelae [1]. Historically, the incidence of bile leakage after open cholecystectomy ranged between 0.17% and 0.5% in large series [2–4]. During the 1990s, higher rates of bile leaks were reported following laparoscopic cholecystectomy, compared to open surgery, reaching up to 3% in some studies [1, 2, 5]. More recent studies, however, have shown substantially lower rates, 0.1% and 0.18% [6, 7]. Based on an analysis of 307,788 cases, Pucher et al. demonstrated a significant decline in bile duct injury rates over time, from 0.69% (1994–1999) to 0.22% (2010–2014) [8].

Laparoscopic cholecystectomy is now the standard surgical treatment for gallstone disease and is one of the most commonly performed abdominal operations by general surgeons [7]. Given the clinical importance of bile duct injuries, critical safety principles have been defined to reduce their occurrence [9, 10]. The management of these injuries is complex and requires a multidisciplinary approach tailored to injury severity and location.

Although several classification systems have been proposed, they often fail to work in many cases and do not typically lead to a therapeutic approach. Existing classifications frequently combine acute and chronic aspects of biliary tract injuries. We hypothesize that existing classification systems are inadequate for the comprehensive categorization of all acute bile duct injuries. The present study aimed to evaluate published classification systems and determine their applicability to all types of bile duct injuries documented using data of the German Arbitration Board. Based on these results, a new classification system was developed.

## Materials and methods

### Database

The Arbitration Board for Medical Liability Issues of the North German State Medical Associations (“Schlichtungsstelle für Arzthaftpflichtfragen der norddeutschen Landesaerztekammern”) was established in 1990. Ten of the sixteen German federal state medical associations participated, including the federal states of Berlin, Brandenburg, Mecklenburg-Western Pomerania, Saxony-Anhalt, Saxony, Thuringia, Schleswig-Holstein, Lower Saxony, Bremen, Hamburg, and Saarland. The arbitration boards provided an out-of-court, independent medical assessment of suspected treatment errors in both inpatient and outpatient care across all medical disciplines. The procedure was free of charge for patients and their relatives, facilitating an out-of-court settlement in cases of proven treatment errors. A subsequent judicial evaluation is generally possible. The Arbitration Board ceased operations in 2021, after which arbitration procedures were transferred to the individual state medical associations.

A total of 9,500 applications were submitted to the Arbitration Board. The Board’s database utilizes the ICD-10 (German Modification International Classification of Diseases) diagnostic codes. All cases with the diagnosis code K80 (cholelithiasis) were extracted from the database (*n* = 345).

For each case, patient files, including medical records, operative reports, expert opinions, and arbitration decisions, were reviewed. Among the 345 patients with cholelothiasis, 106 cases of bile duct injury following cholecystectomy were identified. For these patients, the following data were recorded: age, gender, surgical indication, operative duration, surgical procedure, symptoms, diagnosis and treatment of bile duct injury, localization of the bile duct injury, associated vascular injuries, and complications. The cases were evaluated to determine their suitability for classification using both the published classifications and the newly developed systematic Brandenburg classification.

Ethical approval was obtained from the ethics committee of Brandenburg Medical School (E-01-20190607 / 2019). Statistical analysis was performed using chi-square test of fit with continuity correction to assess the distribution of frequencies. A *p*-value < 0.05 was considered statistically significant.

### Published classifications of bile duct injuries

A narrative literature search was conducted in Medline, PubMed, and Embase using the term [bile duct injury AND classification]. This search yielded 284 publications, of which 59 specifically proposed or applied classification systems for bile duct injuries. Additional references were identified by manual review of key articles. Only publications in English or German were included and the scope was limited to classifications of bile duct injuries following cholecystectomy for benign disease. In total, 11 distinct classification systems were identified [11–23]. Karanikas et al. provided an overview of the published classifications in 2016 [1].

### New systematic classification of bile duct injuries

Brandenburg Acute Bile Duct Injury Classification (BABD Injury Classification).

**Fig. 1:**
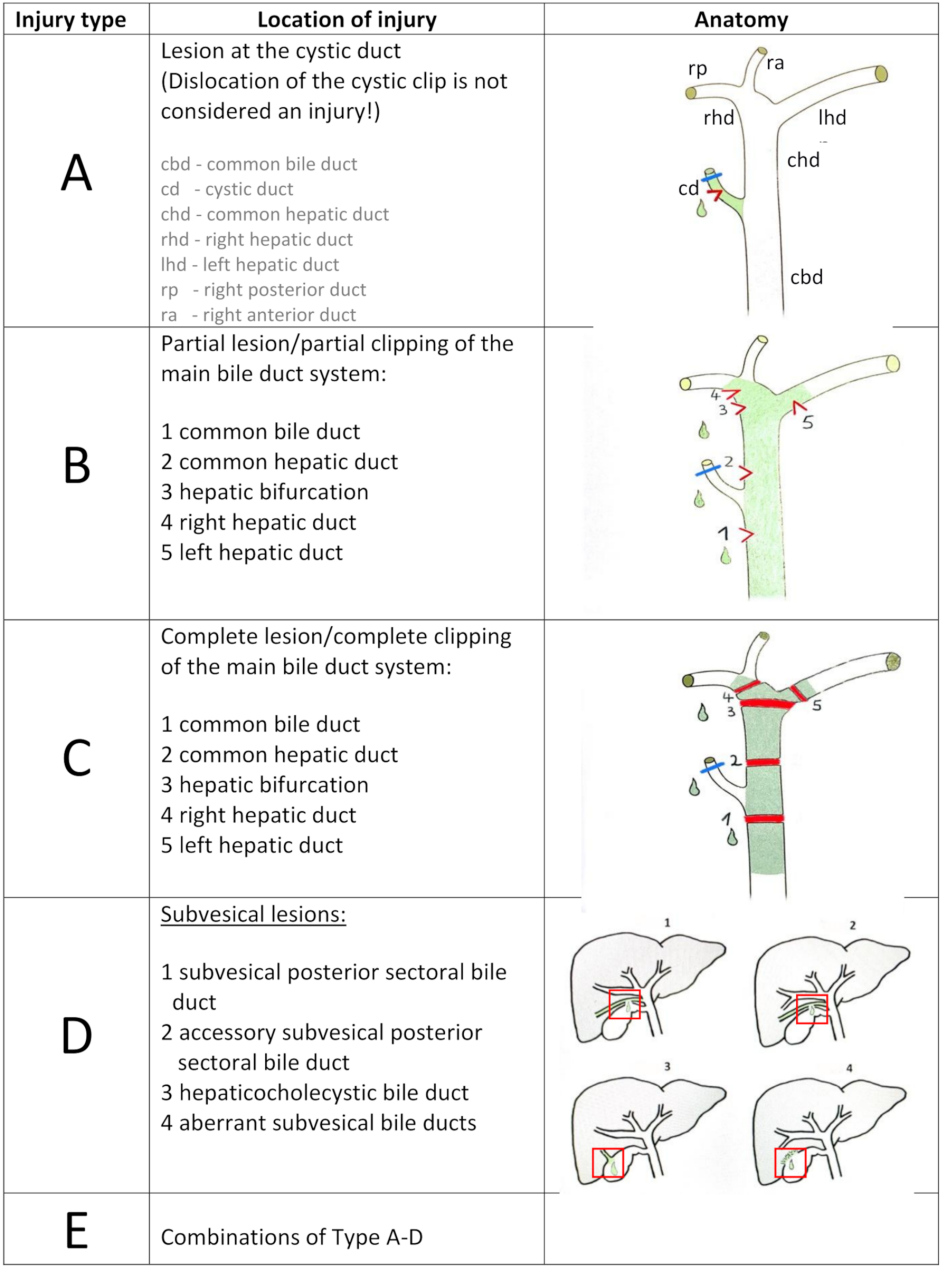
Brandenburg Acute Bile Duct Injury Classification, a vascular lesion will be added with + and the name of the vessel (rha, lha, cha, pv), figure **D** modified from Schnelldorfer et al. 2012, figure **A**, **B**, **C** modified from Mantke, risks and complications in general and visceral surgery, © Thieme, Germany 2018. This material is exempted from this open access license and any further reuse will require separate permission from permission@thieme.de

To address the limitations of existing systems, we developed a new systematic classification based on both the anatomical location and the extent of injury (Fig. 1). The system defines cystic duct injuries (Type A), partial injuries of the common bile duct system (Type B), complete transsections of the common bile duct system (Type C), and subvesical bile duct injuries (Type D) [24]. Type E describes combined injuries involving Types A-C. The localization of the damage is specified numerically using numbers that correspond to the anatomical level of the injury, while associated vascular injuries are indicated by abbreviations of the anatomical designations (Fig. 1). Examples include B3 (partial lesion of the hepatic bifurcation) and C2 + rha (complete transsection of the common hepatic duct with concomitant right hepatic artery injury). The objective was to develop a classification system capable of systematically categorizing all acute bile duct lesions.

## Results

### Patient characteristics

A total of 106 patients with bile duct injuries were analyzed. Patient age ranged from 13 to 83 years, with a meean 49.4 years (SD 16.0). Women were more frequently affected than men (*n* = 67, 63.2% vs. *n* = 39, 36.8%). Only one cholecystectomy was performed as a primary open procedure. Thus, 99% (*n* = 105) of the injuries occurred during laparoscopic surgery. In 37 patients (35.2%), conversion from laparoscopic to open surgery was documented. The mean operative duration was 126.5 min (SD 79.9), ranging from 35 to 365 min. Surgical indications included symptomatic gallstones (69.8%), acute cholecystitis (19.8%), chronic cholecystitis (5.7%), asymptomatic gallstones (3.8%), and Mirizzi syndrome (0.9%).

### Injured structures, time of diagnosis, and therapy

**Fig. 2:**
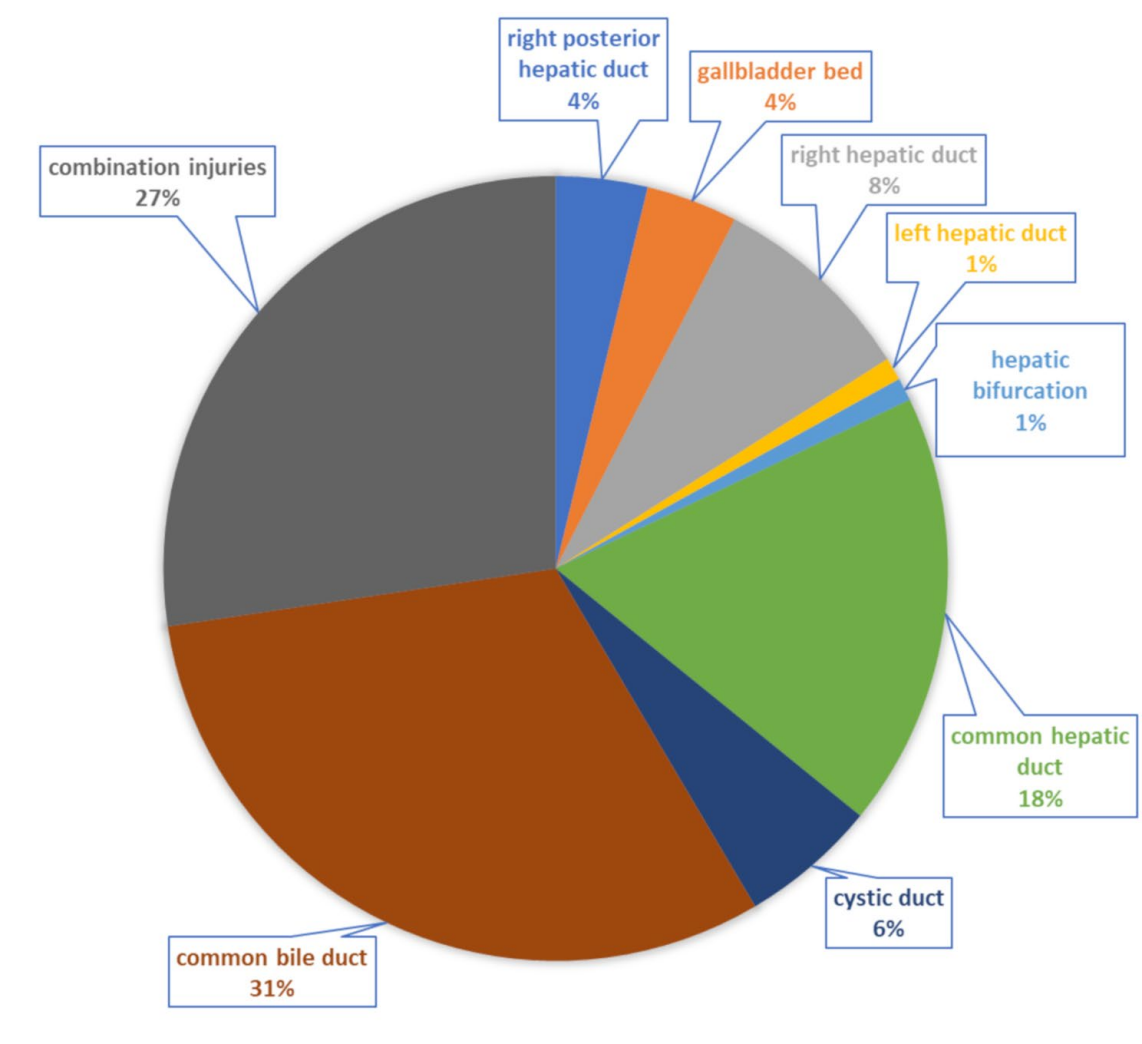
Localization of the bile duct injury after laparoscopic cholecystectomy (*n* = 106)

The common bile duct was the most frequently injured structure (31%) (Fig. 2). Combined bile duct injuries were observed in 27% of the patients, and the common hepatic duct was affected in 18%. Injuries to the left hepatic duct and the hepatic bifurcation were the least common.

Concomitant vascular injury was identified in 13.2% of cases (*n* = 14). The right hepatic artery was injured in 13 cases (93% of all vascular injuries), while the common hepatic artery and the right portal vein were each injured once. The latter was injured in association with the right hepatic artery. Half of the arterial injuries (50%, *n* = 7/14) occurred in compound bile duct injuries. Vascular injuries occurred in combination with an injury to the common hepatic duct in 28.6% (*n* = 4/14) of cases. Arterial injuries occurred alongside a common bile duct injury in 21.4% (*n* = 3/14) of cases (Table 1).

**Table 1:**
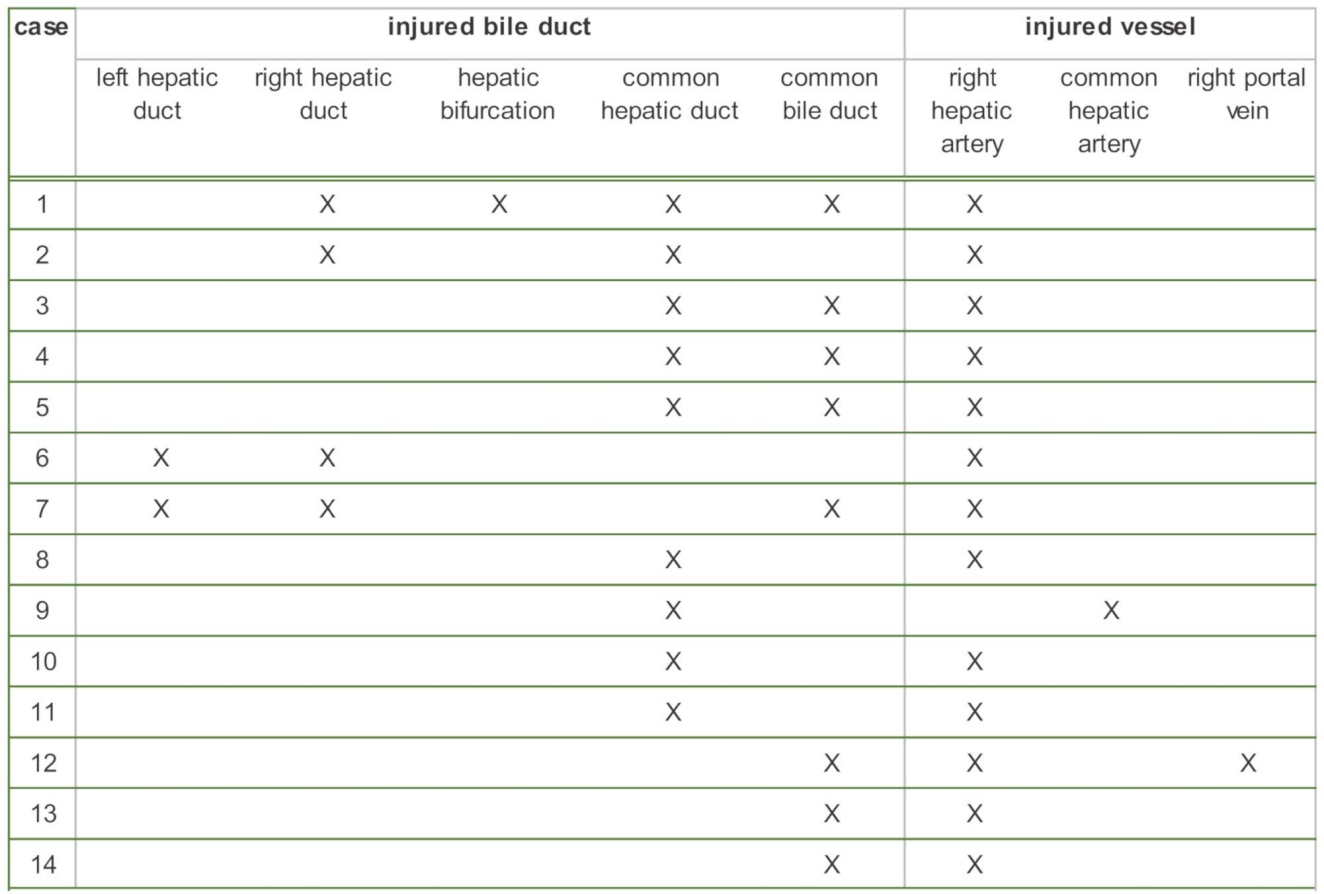
Bile duct injuries and their combination with vascular injuries (*n* = 14)

Bile duct injury was detected intraoperatively in 30.2% of cases (*n* = 32, primary therapy group), whereas 69.8% (*n* = 74) were diagnosed postoperatively (secondary therapy group) (Table 2). In the primary therapy group, initial treatment during the index cholecystectomy was insufficient in 56% of cases, necessitating revision surgery. Univariate analysis of age, sex, surgical indication, and hospital level of care showed no significant association with the likelihood of intraoperative detection (Table 3).

**Table 2:** Timing of detection of bile duct injury: intraoperative versus postoperative (*n* = 106)

injured bile duct	time of detection	
	intraoperatively	postoperative
right posterior bile duct	25% (*n* = 1)	75% (*n* = 3)
gallbladder bed	0	100% (*n* = 4)
cystic duct	0	100% (*n* = 6)
common hepatic duct	42,11% (*n* = 8)	57,89% (*n* = 11)
common bile duct	54,55% (*n* = 18)	45,45% (*n* = 15)
hepatic bifurcation	0	100% (*n* = 1)
left hepatic duct	0	100% (*n* = 9)
right hepatic duct	0	100% (*n* = 1)
combination injuries	12,24% (*n* = 5)	82,76% (*n* = 24)
**total**	**30**,**19%** (***n*** = **32**)	**69**,**81%** (***n*** = **74**)

**Table 3:** Univariate analysis of the risk of not detecting bile duct injury during cholecystectomy

Characteristic	Overall *N* = 106^1^	intraoperative *N* = 32^1^	postoperative *N* = 74^1^	*p*-value^2^
Age				0.695
Mean (SD)	49.4 (16.0)	50.9 (16.7)	48.8 (15.8)	
Median (IQR)	48.5 (39.0–61.0)	50.0 (39.0–64.0)	48.0 (38.0–60.0)	
Sex				0.329
Male	39 (100.0%)	14 (35.9%)	25 (64.1%)	
Female	67 (100.0%)	18 (26.9%)	49 (73.1%)	
Indication				0.321
Acute cholecystitis	21 (100.0%)	5 (23.8%)	16 (76.2%)	
Asymptomatic gallstones	4 (100.0%)	1 (25.0%)	3 (75.0%)	
Chronic cholecystitis	6 (100.0%)	4 (66.7%)	2 (33.3%)	
Mirizzi syndrome	1 (100.0%)	0 (0.0%)	1 (100.0%)	
Symptomatic gallstones	74 (100.0%)	22 (29.7%)	52 (70.3%)	
Hospital care level				0.563
Basic care	10 (100.0%)	2 (20.0%)	8 (80.0%)	
Standard care	56 (100.0%)	17 (30.4%)	39 (69.6%)	
Specialized care	32 (100.0%)	9 (28.1%)	23 (71.9%)	
Maximum care	8 (100.0%)	4 (50.0%)	4 (50.0%)	
Hospital teaching status				0.450
Teaching	99 (100.0%)	29 (29.3%)	70 (70.7%)	
Non-teaching	7 (100.0%)	3 (42.9%)	4 (57.1%)	
University hospital				0.163
Yes	3 (100.0%)	2 (66.7%)	1 (33.3%)	
No	103 (100.0%)	30 (29.1%)	73 (70.9%)	

^1^n (%)

**Fig. 3:**
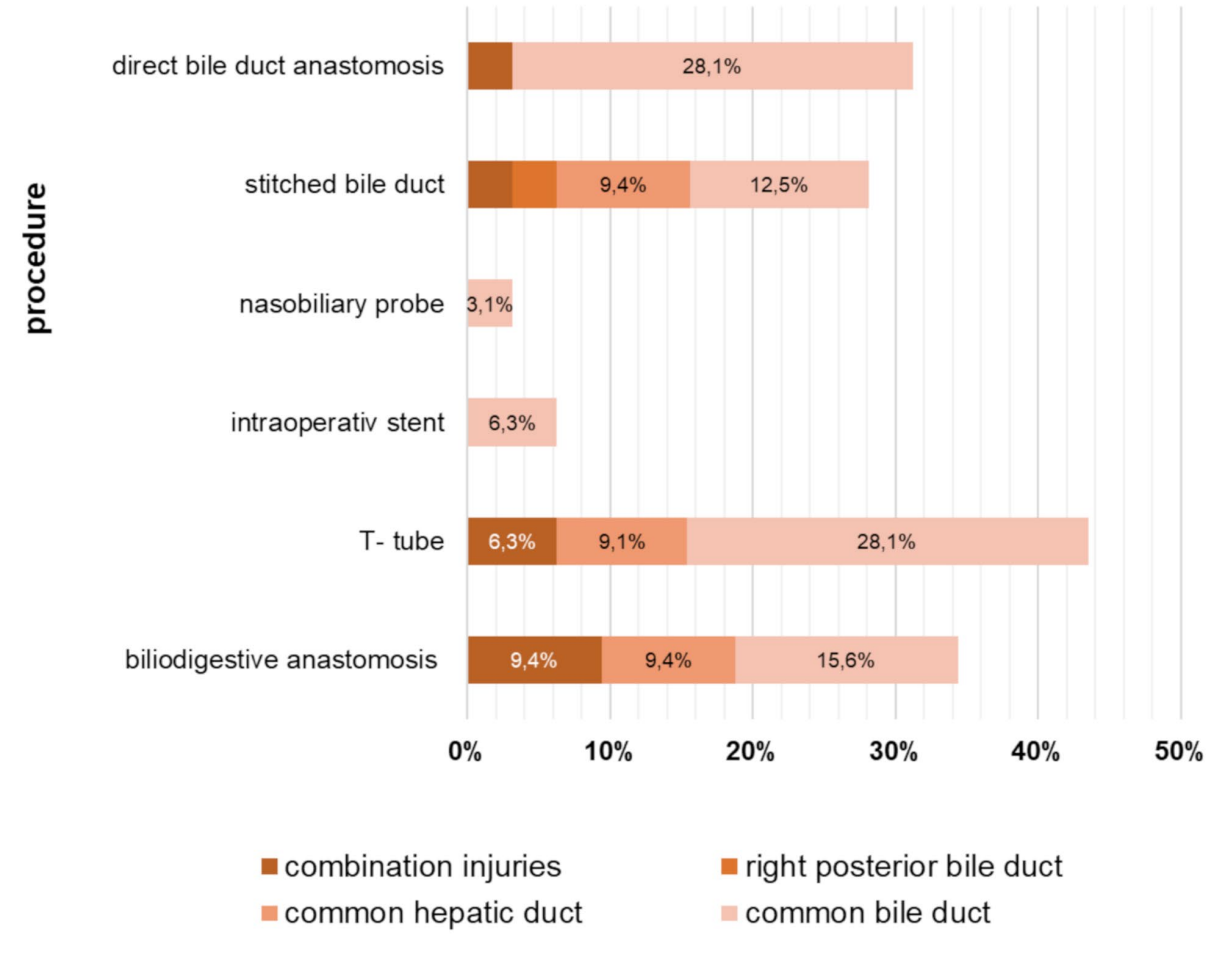
Therapy methods for bile duct injuries detected intraoperatively during cholecystectomy: A combination of procedures is possible (*n* = 32)

**Fig. 4:**
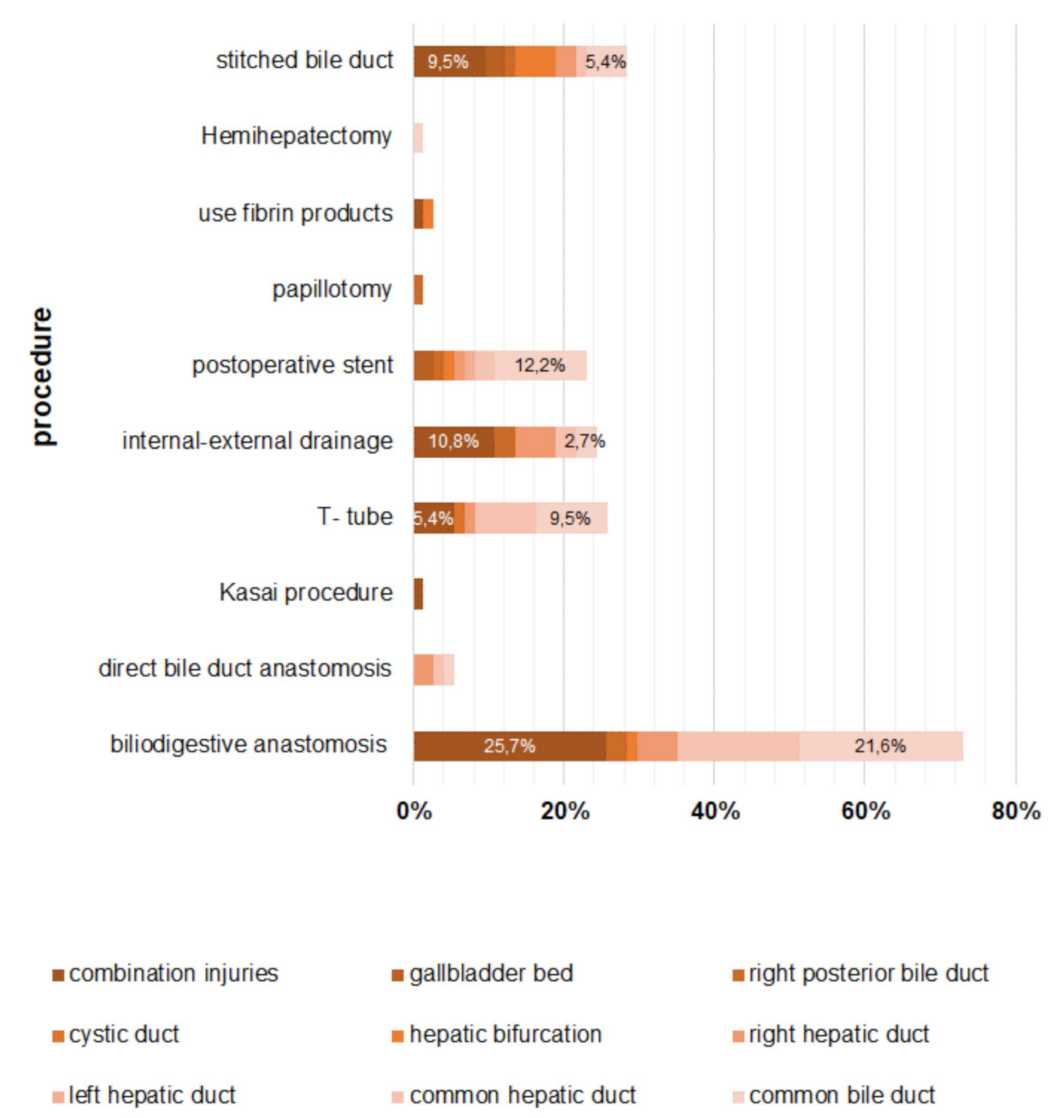
Operative and conservative therapy methods for bile duct injuries diagnosed postoperatively: A combination of procedures is possible (*n* = 74)

In the secondary therapy group, 71 patients underwent reoperation, while 3 patients were successfully treated with nonsurgical interventions (A, D1, and B5 according to the BABD Classification). Surgical repair techiques of the bile ducts differed makedly between therapy groups (Figs. 3 and 4). In the primary therapy group, the most frequent procedures were bile duct anastomosis (31.3%), closure of the leak with stitches (28.1%), and biliodigestive anastomosis (34.4%, typically as a Roux-en-Y hepaticojejunostomy). In the secondary therapy group, biliodigestive anastomosis was most frequently used (73%), followed by closure of the leak with stitches (28.4)%, and direct bile duct anastomosis (5.4%). Stenting of the bile duct was more common in the secondary therapy group (23.0% versus 6.3%), whereas T-tube insertion was more frequently applied in the primary surgical therapy group (43.8% versus 25.7%). Internal-external drainage as a splint for bile duct anastomosis was used in 24.3% of secondary operations.

### Mortality

Four patients (3.8%) died as a direct consequence of bile duct injury. This included three common bile duct injuries and one combination injury with lesions at different sites of the bile duct system (2 x C1, 1 x C1rha + pv, 1 x E, according to the BABD Classification). Two bile duct injuries were detected intraoperatively, while 2 were diagnosed postoperatively. In the four patients who died, the bile duct lesion was overstitched once, a bile duct anastomosis with a T-tube insert was performed once, and a hepaticojejunostomy was performed twice. All four patients died of multi-organ failure due to the inability to control the bile duct lesion.

### Application of different bile duct injury classification systems

All 106 cases of bile duct injury were evaluated using previously published classification systems. The number of classifiable cases is summarized in Table 4. None of the published systems was able to classify more than 90% of bile duct injuries. The highest categorization rates were achieved by the Amsterdam and Hannover systems, with rates of 85.9% and 82.1%, respectively. Unclassifiable injuries in both systems involved lesions of anatomical structures of the common hepatic duct and the common bile duct, or were combination injuries. Even the widely used Bismuth system was applicable to only 77.3% of bile duct injuries.

**Table 4:**
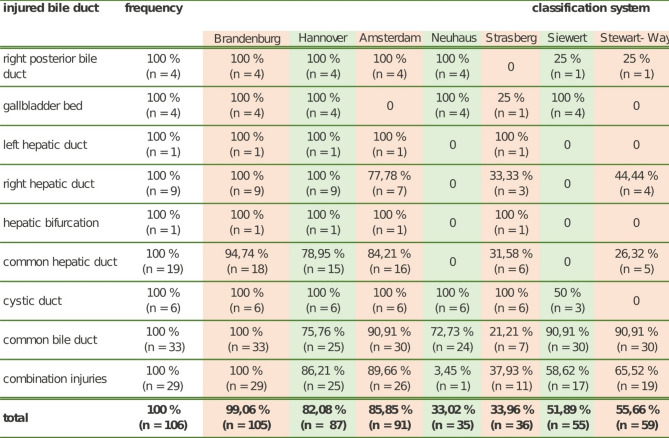
Use of various published classification systems for bile duct injuries and the newly developed Brandenburg classification (Brandenburg acute bile duct injury classification) after cholecystectomy: how many cases (%) could be classified with different classification systems? (*n* = 106)

By contrast, the Brandenburg Acute Bile Duct Injury Classification system was applicable to nearly all injury patterns (99%). For 105 out of 106 cases, it was possible to use the Brandenburg classification (Table 4). One case remained uncategorized due to insufficient documentation in the medical records about the injury. Most of the injuries were complete lesions or clippings of the common bile duct system and were classified as Type C (*n* = 42, 40%) (Table 5). The observed frequencies of the various injuries differed significantly (*p* < 0.001), with Type C injuries being the most frequent (*p* < 0.001). Combination injuries (Type E) were the second most common category (*n* = 29, 27.6%). Concomitant vascular injuries varied across categories, occuring in 0% of Type A, 5% of Type B, 14.3% of Type C, 0% of Type D, and 24.1% of Type E, relative to the total number of cases within each type.

**Table 5:** Frequency of bile duct injury type, frequency of additional vascular injury, and biliodigestive anastomosis using the Brandenburg Acute Bile Duct injury classification

Type	case number	frequency	case number vascular injury	frequency vascular injury	case number biliodigestive anastomosis	frequency biliodigestive anastomosis
A	6	5,7%	0	0%	0	0%
B	20	19%	1	5%	3	15%
C	42	40%	6	14,3%	32	76,2%
D	8	7,6%	0	0%	2	25%
E	29	27,6%	7	24,1%	20	69%

## Discussion

The incidence of bile duct injuries during laparoscopic cholecystectomy remains a subject of ongoing discussion. In a large analysis conducted in 2018, Pucher et al. reported a decline in incidence to 0.22% over the past decade in a substantial population [8]. This rate is consistent with historical data for open cholecystectomy [2–4]. Despite expanded indications for laparoscopic cholecystectomy, it can now be asserted that the procedure no longer carries an increased risk of bile duct injuries, provided that the criteria for safe laparoscopic cholecystectomy, as defined by expert consensus, are implemented in clinical practice [9, 10].

Numerous classifications of bile duct injuries have been proposed [1, 11–15, 17–23]. In our view, these classifications are not entirely practical for clinical use. Bile duct injuries are often complex, and not all cases can be adequately categorized using existing systems. Furthermore, many classifications conflate acute injuries with their chronic sequelae. The advantages and limitations of these classifications have been extensively discussed [1, 11, 15]. In this study, we applied all relevant published classifications to a multicenter patient population with bile duct injuries following laparoscopic cholecystectomy. We analyzed 106 cases of iatrogenic bile duct injuries from the North German Arbitration Board database for medical liability issues. It is important to note that only cases where patients sought to verify potential treatment errors were included, consequently our cohort represents a selection of serious complications after laparoscopic cholecystectomy. The sample size is comparable to that of other studies on bile duct injuries [11, 18]. Unlike previous studies, our study utilizes a multicenter setting.

The observed mortality rate of 3.8% is within the range reported by other studies (3–11%) [1, 11, 25, 26]. Death typically resulted from insufficient repair of the bile duct injury, leading to persistent peritonitis, sepsis, and multi-organ failure. To prevent mortality, early diagnosis and professional management are crucial. For these reasons, we advovate that patients with complex iatrogenic bile duct injuries (BABD classification Type C and E) should be transferred to a center with extensive experience in bile duct surgery, if such expertise is not available at the primary hospital. This is particularly important as the treatment of bile duct lesions is often interdisciplinarya and surgical expertise alone is insufficient. A comprehensive approach, ranging from computed tomography (CT)-guided drainage to various endoscopic and surgical techniques, is required [27].

Our study provides a systematic overview of bile duct injury locations and the frequency and location of associated vascular injuries. The right hepatic artery is particularly at risk due to its close anatomical proximity to the operative field during cholecystectomy (12.3% right hepatic artery injuries in our cohort). This risk has been similarly described by other authors [11, 25]. Therefore, we always recommend an abdominal CT scan with arterial and portal vascular imaging prior to surgical revision following complicated cholecystectomy.

We found that only 30.2% of bile duct injuries were detected intraoperatively during the initial cholecystectomy. This compares to an intraoperative detection rate of 36% reported by Bektas et al. and 5% by Balla et al. [11, 25]. This indicates that in 70% of cases, the surgeon is unaware of the intraoperative error. This likely occurs either because the established criteria for safe laparoscopic cholecystectomy were not met or because the anatomical conditions were not correctly assessed. Therefore, it is important not only to adhere to defined safety criteria during gallbladder removal but also to train surgeons to better recognize intraoperative bile duct injuries. A intraoperatively identified injury can be treated appropriately immediately. If this is not possible due to a lack of experience in the team, the area should be drained, and the patient promptly transferred to a specialized center. The World Society of Emergency Surgery has developed guidelines for the detection and management of bile duct injuries during cholecystectomy [27].

We were unable to identify risk factors for intraoperatively overlooked bile duct lesions in our patient population, as they occurred at all levels of hospitals.

While over-sewing of the defect, T-tube drain insertion, direct bile-duct anastomosis, and biliodigestive anastomosis were used with similar frequency in the primary treatment, a biliodigestive anastomosis was performed in 74% of revision cases. This difference may reflect avoidance or limited expertise with biliodigestive anastomosis during the index procedure. However, it is also possible that with secondary treatment, tissue damage and inflammation are more extensive than immediately after the injury, necessitating more tissue debridement before treating the defect. Consistent with previous reports, direct suture is more frequently used than a Roux-en-Y anastomosis in cases of primary detection of bile duct injury [7].

The rate of postoperative bile duct strictures in this study was 11.7% (2 of 19 treated patients), which can be considered acceptable [7]. However, primary management failed in 56% of cases, requiring reoperation. These findings suggest that biliodigestive anastomosis should be considered more often in the primary management of complex bile duct injuries. Although follow-up data were not available in this study, Roux-en-Y anastomosis is recognized as a safe surgical procedure with good long-term outcomes, albeit with a potential increased risk of cholangitis over time [28, 29].

Pekolj et al. demonstrated that routine use of intraoperative cholangiography can increase the detection rate of bile duct injuries to 90% in over 10,000 cases [7]. Accordingly, we advocate a liberal indication for intraoperative cholangiography, particularily in cases of anatomical peculiarities or unclear structures in the Calot’s triangle. Bile duct visualization should be performed to prevent bile duct injuries or increase the proportion of injuries detected intraoperatively. The use of ICG technology (indocyanine green fluorescence cholangiography) for visualizing bile duct anatomy is increasingly discussed in the literature as an alternative [27]. However, the results and the level of evidence still need evaluation. Additional surgical strategies to avoid bile duct injuries include the fundus-first approach (top-down), subtotal cholecystectomy (bailout procedure), or early conversion to open cholecystectomy [27].

Several classification systems for bile duct injuries have been published [11–23, 30], with Bismuth and Strasberg classifications being the most frequently used. The Bismuth classification, introduced in 1982, was primarily developed to classify biliary strictures [16]. The Strasberg classification, published in 1995, is an extension of the Bismuth system, designed to incorporate acute bile duct injuries [23, 31].

Strasberg’s classification primarily addresses biliary strictures, and not all injury patterns or combination injuries can be categorized using either the Bismuth or Strasberg systems. Consequently, both classifications are inadequate for classifying acute bile duct injuries, as they do not support scientific comparisons or guide therapeutic decision making. Similarily, the Hanover, Neuhaus, Amsterdam, Stewart-Way, and Siewert classifications incorporate late-onset strictures, limiting their utility in acute injury scenarios. Given the complexity of biliary duct injuries, a classification system specifically focused on acute injuries is required, particularly for classify complex injuries, concomitant hepatic artery injuries, or subvesical bile duct lesions, which cannot be reliably categorized using the published classifications.

Bile duct injuries can be classified according to their anatomical location and/or extent of the lesion (complete vs. partial transection). The EAES classification, published in 2016, is comprehensive and integrate elements of all previously published systems [15]. However, its complexity limits its practical application in routine clinical practice. In our analysis, none of the published systems could classify all cases. The Hanover and Amsterdam systems achieved the highest applicability at 82% and 85%, respectively [11, 12].

In contrast, the Brandenburg Acute Bile Duct Injury Classification system enables systematic categorizations of all acute bile duct injuries. This facilitates both diagnostic and therapeutic descision-making and supports standardized scientific analyses that are easily comparable across studies (Table 6).

**Table 6:** Diagnosis and therapy for postoperatively detected bile duct injuries based on the Brandenburg Acute Bile Duct injury classification

Type Brandenburg Classification	Diagnostic / Conservative / Interventional therapy	Conservative / Interventional therapy	Surgery
**A**	MRC, Endoscopic retrograde cholangiography	External drainage, endoscopic stenting	Only if interventional therapy does not work
**B**	MRC, Endoscopic retrograde cholangiography, computed tomography	For small lesions, external drainage and endoscopic stenting	For larger lesions, surgical revision is necessary, with a preference for biliodigestive anastomosis
**C**	MRC, Endoscopic retrograde cholangiography, computed tomography	-	Surgical revision, with a preference for biliodigestive anastomosis
**D**	MRC, Endoscopic retrograde cholangiography	External drainage, endoscopic stenting	If interventional therapy is not successful, surgical revision and over-stitching the lesion; note that in D1 and D2 lesions, this can lead to dilatation and obstruction of the right posterior segments
**E**	MRC, Endoscopic retrograde cholangiography, computed tomography	-	Revision, with a preference for biliodigestive anastomosis

Our study has several limitations. It included only patients with suspected treatment errors and sought arbitration board evaluation, likely representing a selection of particularly severe cases. Consequently, bile duct injuries managed solely with postoperative interventional treatment may be underrepresented. The available arbitration board files, while sufficient for expert assessment, did not always contain complete medical records. Finally, our study does not provide data on the overall incidence of bile duct injuries.

## Conclusion

Existing classification systems for bile duct injuries do not allow complete categorization of all bile duct injuries after cholecystectomy. In our analysis of 106 patients, none of the 11 published systems achieved full applicability. In contrast, the newly developed Brandenburg Acute Bile Duct Injury (BABD) Classification enabled systematic categorization of all documented acute lesion, including complex and combined injuries.

A standardized and universally applicable classification is essential to improve clarity in clinical communication, ensure comparability of outcomes across studies, and guide therapeutic decision-making. The BABD Classification provides a structured framework that can be integrated into clinical practice and research to support consistent reporting and management of bile duct injuries.

## Data Availability

The datasets used and analysed during the current study are available from the corresponding author on reasonable request.
